# How Are Proteins Reduced in the Endoplasmic Reticulum?

**DOI:** 10.1016/j.tibs.2017.10.006

**Published:** 2018-01

**Authors:** Lars Ellgaard, Carolyn S. Sevier, Neil J. Bulleid

**Affiliations:** 1 Department of Biology, University of Copenhagen, 2200 Copenhagen, Denmark; 2 Department of Molecular Medicine, College of Veterinary Medicine, Cornell University, Ithaca, NY 14853-2703, USA; 3 Institute of Molecular, Cell and Systems Biology, College of Medical, Veterinary and Life Sciences, University of Glasgow, Glasgow G12 8QQ, UK

**Keywords:** protein folding, ER-associated degradation, ER chaperones, thiol reduction, endoplasmic reticulum, disulfides

## Abstract

The reversal of thiol oxidation in proteins within the endoplasmic reticulum (ER) is crucial for protein folding, degradation, chaperone function, and the ER stress response. Our understanding of this process is generally poor but progress has been made. Enzymes performing the initial reduction of client proteins, as well as the ultimate electron donor in the pathway, have been identified. Most recently, a role for the cytosol in ER protein reduction has been revealed. Nevertheless, how reducing equivalents are transferred from the cytosol to the ER lumen remains an open question. We review here why proteins are reduced in the ER, discuss recent data on catalysis of steps in the pathway, and consider the implications for redox homeostasis within the early secretory pathway.

## Trends

Correct disulfide formation within the secretory pathway requires both disulfide bond formation and disulfide reduction.

Protein thiol modification is reversed by protein disulfide isomerase (PDI) family members localized to the ER.

A pathway links thioredoxin reduction in the cytosol to disulfide reduction in the ER.

Misfolded ER proteins need to be reduced before targeting for destruction in the cytosol.

The unfolded protein response (UPR) transducers Ire1 and ATF6 are regulated by disulfide bond formation and reduction.

The key regulator of protein folding and the UPR, BiP, is modulated by reduction catalyzed by the non-canonical oxidoreductase Sil1.

## Why Is Protein Reduction Important?

**Figure 1:**
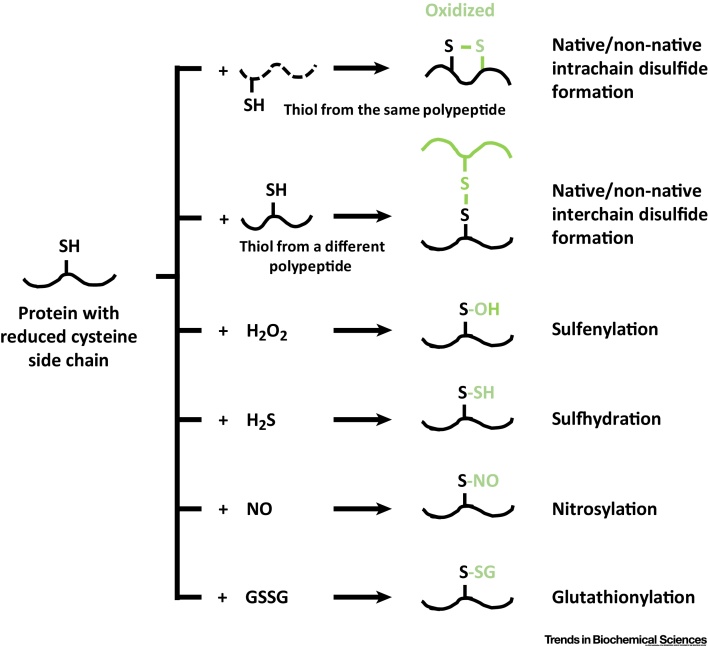
Pathways for Oxidation of Protein Thiols. Oxidation of a protein thiol can result in several different outcomes depending upon the type of oxidant. We depict here the formation of intra- or inter-chain disulfides with small-molecule oxidation by hydrogen peroxide or sulfide, nitric oxide, or glutathione disulfide. In each case the modification to the thiol group is depicted in green.

Most thiols in secretory proteins are modified as they enter the ER (Figure 1). The majority form disulfide bonds between parts of the same polypeptide or between different chains. During protein folding, disulfides can form that are not present within the final native structure. Such non-native disulfides are prevalent in misfolded proteins, but can also form as part of the normal folding pathway [1]. Reduction of these disulfides is crucial for correct folding and for degradation of misfolded proteins. The importance of a reductive pathway to remove non-native disulfides is exemplified by cells that produce large amounts of disulfide-bonded proteins, such as insulin in pancreatic β cells or antibodies in plasma cells. The accumulation of non-native disulfide-bonded insulin following glucose stimulation can lead to loss of insulin secretion, oxidative stress, and apoptosis, resembling pathologies seen in type II diabetes [2]. Similarly, aggregation occurs when the accumulation of misfolded immunoglobulins exceeds the cellular capacity to remove aberrantly disulfide-bonded proteins from the ER [3].

**Figure 2:**
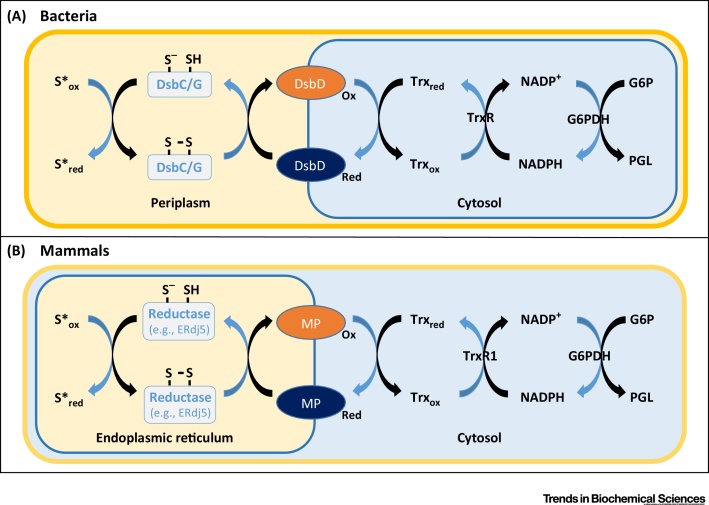
Reducing Equivalents from the Cytosol Sustain Protein Reduction in the Periplasm and the Endoplasmic Reticulum (ER). (A) In bacteria, oxidized (ox) thiols (S*) in periplasmic proteins are reduced (red) by DsbC (disulfides) or DsbG (sulfenylated thiols), which in turn are reduced by the membrane protein DsbD. DsbD is maintained in a reduced state by the cytosolic thioredoxin system, with the ultimate electron donor being NADPH. (B) In the mammalian ER non-native disulfides can be reduced by the cytosolic reductive pathway involving thioredoxin and thioredoxin reductase. The ultimate electron donor in the pathway is NADPH (on the right) that can be generated by glucose 6-phosphate dehydrogenase (G6PDH) during the conversion of glucose 6-phosphate (G6P) to phosphogluconolactone (PGL). NADPH is required by thioredoxin reductase (TrxR1) to drive the reduction of thioredoxin (trx). The link between reduced thioredoxin in the cytosol and the reduction of an ER oxidoreductase such as ERdj5 is unknown but is most likely a membrane protein (MP).

In addition to the reduction of non-native disulfides, there is a requirement to reduce cysteine side chains modified by small molecules. These molecules include hydrogen peroxide, hydrogen sulfide, nitric oxide, and glutathione 4, 5, 6 which mediate sulfenylation, sulfhydration, nitrosylation, and glutathionylation respectively (Figure 1). Oxidation of cysteine side chains can be a mechanism for regulating protein function or for signaling. For some enzymes, the recycling of oxidized active-site cysteines to the thiol form is essential to maintain activity 7, 8. Several recent studies have provided examples of proteins that are susceptible to such modification, and have determined the consequences of cysteine modification for protein function 9, 10, 11, 12, 13. Reduction of these modified thiols in the ER is likely to be catalyzed by an oxidoreductase such as a protein disulfide isomerase (PDI) family member. There is precedent for such a reductive pathway in bacteria [10] and in yeast and mammalian cytosol 9, 12. In the bacterial periplasm correct disulfide formation requires both an oxidative and a reductive pathway. For the reductive pathway, a soluble periplasmic protein DsbC/G catalyzes the initial reduction of oxidized thiols, and then passes its disulfide to the plasma-membrane protein DsbD. The disulfide is then shuttled across the membrane by internal disulfide exchange and is ultimately reduced by the cytosolic thioredoxin/thioredoxin reductase pathway (Figure 2A). The fact that disulfide exchange proteins within the ER require their active-site disulfide to be reduced to maintain reductase activity has stimulated the search for components of the reductive pathway and the identification of the ultimate electron donor.

## How Are Non-Native Disulfides Reduced to Allow Protein Folding?

The initial step in the reduction pathway is catalyzed by ER oxidoreductases, the most abundant of which are members of the PDI family (Box 1). To catalyze reduction, the active-site cysteine pair must be capable of efficiently donating electrons to its substrate protein. The type of reaction, oxidation or reduction, that is catalyzed by individual members of the PDI family is to some extent determined by the stability of the disulfide formed within its active site [14]. This stability is determined by the electron-withdrawing effect of nearby amino acids that differ between members of the PDI family. The more stable the disulfide, the more likely the enzyme will act as a reductase and donate electrons. Conversely, the less stable the disulfide, the more likely it will act as an electron acceptor and catalyze oxidation. Hence, not all PDIs are equivalent; their active-site disulfide(s) have different stabilities, and this contributes toward the propensity for some PDIs to act primarily as reductases and others to function in oxidation.

Box 1 **Key Features of the Protein Disulfide Isomerase Family**

Disulfide bond formation in the ER is catalyzed by the PDI family of dithiol-disulfide oxidoreductases [80]. About 20 proteins have been assigned to this family to date, with a minimum of 15 members having at least one domain containing the typical thioredoxin-like fold with a characteristic CXXC motif at the active site. These proteins catalyze thiol–disulfide exchange reactions by acting as electron acceptors during disulfide bond formation (oxidation) or as electron donors during breaking of disulfides (reduction). The PDI proteins also catalyze isomerization reactions by rearranging non-native to native disulfides. The functions of some of the PDI proteins have been thought to be redundant because of the similarities in their active sites. However, there is now accumulating evidence for separate substrate specificities and defined functions either as oxidases or reductases [81].

PDI was the first ER oxidoreductase to be extensively characterized. It is an essential enzyme in yeast, and has been shown not only to introduce disulfide bonds into substrates but also to function as a chaperone 82, 83. PDI contains four thioredoxin-like domains namely a, b, b′, and a′. The a and a′ domains contain canonical CXXC active-site motifs, with redox potentials of between −160 and − 170 mV [20]. The low pK_a_ of the first cysteine of the CXXC motif allows this residue to participate in disulfide bond formation [84]. The crystal structure of the yeast and mammalian PDI has been solved. It shows an arrangement of the four PDI domains in a twisted ‘U’ conformation, with the catalytic domains a and a′ being situated at the top, facing each other, while the domains b and b′, are sandwiched between the catalytic domains, on the inside of the ‘U’ [85]. The interior of the U is very hydrophobic, and has been shown to be the principal binding site for peptides and misfolded regions of substrates 86, 87.

When PDI and other oxidoreductases introduce disulfides into newly synthesized proteins, their active-site CXXC sequences must be reoxidized to allow further rounds of disulfide formation. This function is fulfilled by specific ER-resident oxidoreductases which do not directly introduce disulfides into newly synthesized proteins. These enzymes catalyze the first step in disulfide formation by transferring oxidizing equivalents to the PDI proteins, which then introduce these disulfides into nascent polypeptides [26].

Alt-text: Box 1

In addition to disulfide stability determining oxidoreductase activity, it is also important to acknowledge that kinetic factors can influence oxidoreductase functions. For example, the enzyme responsible for oxidation of ER oxidoreductases, ER oxidase 1 (Ero1), preferentially oxidizes PDI over other family members, most likely because of the ability of Ero1 and PDI to physically interact [15]. Any potential reductase may well physically interact with an individual member(s) of the PDI family, thereby providing a kinetic as well as thermodynamic driver to determine reactivity. Such a kinetic segregation ensures that there is a lack of crossover of the oxidative and reductive pathways within the bacterial periplasm, and may well explain how these two opposing pathways can coexist within the eukaryotic ER 16, 17.

The stabilities of some of the active-site disulfides of individual PDIs have been measured experimentally 18, 19, 20, 21. The ERdj5 active sites stand out as being relatively stable, suggesting that this enzyme most likely acts as an ER reductase. Indeed, its role in the reduction of disulfides in proteins during folding and degradation is now well documented 22, 23. For example, depletion of ERdj5 results in a defect in the resolution of non-native disulfides within the low-density lipoprotein receptor (LDLr), leading to a block in its folding and secretion [22]. In addition, another PDI, ERp57, has been shown to be required for the rearrangement of disulfides within glycoproteins, suggesting reductase or isomerase activity [24]. By contrast, PDI itself has a less-stable disulfide and is the primary substrate for oxidation by Ero1, suggesting that its main role is oxidation 15, 25. Thus, a subgroup of PDI enzymes appear to carry out the initial reduction of modified thiols.

### How Are the PDI Family Members Themselves Reduced?

How the active site of PDIs is reduced to allow reductase activity has been the subject of much debate 26, 27. One source of reductant comes from the flux of cysteine thiols that are present in nascent chains translocated across the ER membrane. It is not possible to quantify the number of reducing equivalents introduced by this route, but it is likely to be significant and may well contribute to the redox status of PDI family members. A second abundant potential reductant is reduced glutathione (GSH). Evidence of a role for GSH in reduction has been provided for ERp57, which can be reduced following oxidative stress via a GSH-dependent process 28, 29. However, ERdj5 is mostly oxidized within the ER [30], which is known to have a high ratio of GSH:GSSG [31], suggesting that its active site is resistant to reduction by GSH. Moreover, GSH depletion has no consequences for the formation of correct disulfides in proteins such as LDLr, which is known to require the reduction of non-native disulfides [32], suggesting that the reductase activity of PDIs is not severely impacted by GSH depletion. These apparently conflicting reports concerning the impact of GSH on PDIs may reflect a distinction between the roles of GSH in restoring redox homeostasis and in the resolution of non-native disulfides [33]. In the former, GSH may buffer redox changes indirectly via one of the PDIs or by reacting directly with oxidants. In the latter situation, the non-native disulfide may be too stable for reduction by GSH, and thus requires catalysis by an enzyme such as ERdj5. These observations not only support a role for glutathione as a reductant but also imply a need for a GSH-independent pathway that allows proper folding and the reduction of ERdj5.

A role for the cytosolic thioredoxin system in disulfide formation in the ER has recently been suggested [34]. In the absence of robust recycling of cytoplasmic NADP to NADPH, proteins entering the secretory pathway were shown to form incorrect disulfides when translated in a translation/translocation system. The transition from non-native to native disulfides in the ER was restored upon addition of glucose 6-phosphate (G6-P) to the translation system, which enables efficient recycling of NADP to NADPH via the pentose phosphate pathway. This demonstrated that the generation of cytosolic NADPH allows correct disulfide formation in the ER, and identified NADPH as the ultimate electron donor in the pathway.

G6-P-mediated recovery was prevented when cytosolic thioredoxin reductase (but not glutathione reductase) was inhibited, suggesting that correct disulfide formation in the ER requires the cytosolic thioredoxin reductase system. In addition, inhibition of thioredoxin reductase prevented correct disulfide formation during the synthesis of LDLr in mammalian cells. Together, these data suggest that the correction of non-native disulfides formed in the ER (and PDI family reductase activity) relies on the cytoplasmic NADPH–thioredoxin system.

Because the recycling of NADPH is dependent on the presence of G6-P, disulfide formation in the ER will be affected by changes in glucose metabolism. It has been known for several years that glucose depletion can lead to protein unfolding and induction of the unfolded protein response (UPR) in mammalian cells [35]. This effect was considered to be due to a blockage of protein glycosylation as a result of depletion of precursor sugars for the oligosaccharide chain. The link between glucose metabolism and correct disulfide formation in secreted proteins provides an intriguing alternative explanation.

Linking cytosolic thioredoxin reduction to the reduction of PDIs in the ER would require a so far unidentified membrane protein to transfer reducing equivalents across the ER membrane (Figure 2B). Moreover, it remains an open question how many steps are necessary to deliver the reducing equivalents from the cytosol to the non-native disulfide within the ER. The relative roles of the cytosolic thioredoxin reductase pathway and ER-localized NADPH reductases (see later) are also unknown.

## Is Disulfide Bond Reduction Required for ER-Associated Degradation?

Non-native disulfides are associated with protein misfolding in the ER, and can form within single proteins and between different polypeptide chains to generate large amorphous disulfide-linked aggregates. A longstanding question in the field has been whether disulfide bond reduction is a prerequisite for the degradation of these species.

Proteins that misfold in the ER are degraded by the ubiquitin-proteasome system in the cytosol. Degradation of soluble ER proteins, therefore, requires retrotranslocation across the membrane, whereas ER transmembrane proteins must be extracted from the lipid bilayer. Upon emergence in the cytosol, substrates are ubiquitinated, extracted from the ER by the AAA-ATPase p97, and degraded by the proteasome [36]. This process is termed ER-associated degradation (ERAD).

**Figure 3:**
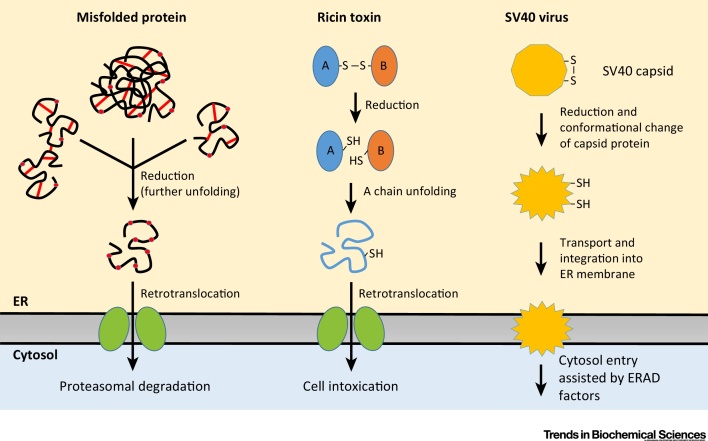
Protein Reduction in Endoplasmic Reticulum (ER)-Associated Degradation (ERAD) and Cytosolic Entry of Toxins and Viruses. (A) Intra- and intermolecular disulfide bonds in misfolded proteins are reduced by members of the protein disulfide isomerase (PDI) family before retrotranslocation. For some proteins, chaperone-assisted unfolding is also likely to occur. Disulfide bonds are drawn as red lines, and free cysteines are shown as red circles. (B) Reduction of an intermolecular disulfide bond between the A and B subunits of toxins such as ricin is required before A-chain unfolding and retrotranslocation. (C) Viruses such as SV40 require isomerization and reduction of a capsid disulfide bond to undergo a conformational change which allows entry of the virus into the cytoplasm.

Analogous to translocation, retrotranslocation has long been assumed to occur via a protein-conducting channel (the ‘retrotranslocon’ or ‘dislocon’). A prime candidate has been Hrd1, a central ERAD E3 ligase. The recent cryo-electron microscopy structure demonstrates that Hrd1 forms a hydrophilic membrane-spanning funnel with a hydrophobic seal forming a membrane barrier near the ER lumen [37]. These overall structural features seem to be conserved in several other E3 ligases involved in ERAD. The Hrd1 structure with its narrow funnel indicates that unfolding is a prerequisite for passage through this (and similar) channels. Concomitantly, disulfide bond reduction is likely a requirement for the degradation of many ERAD substrates. Indeed, various ERAD substrates, as well as viruses and toxins, have been shown to undergo reduction before retrotranslocation 38, 39, 40, 41 (Figure 3). Moreover, oxidizing conditions in the ER can prevent retrotranslocation, whereas reducing conditions tend to promote the process 39, 42, 43. However, it is worth noting that these latter results may not reflect obligate reduction of ERAD substrates, and instead could indicate a role for redox modification of the retrotranslocation machinery. It has been suggested that some ERAD substrates may be able to cross the membrane in a folded state 44, 45, indicating that alternative (non-Hrd1) mechanisms of retrotranslocation may also exist.

### Reductase Function of PDIs during ERAD

Among PDIs, ERdj5 is the most prominent ERAD reductase. It was first identified as an ERAD reductase for the null Hong Kong variant of α1-antitrypsin and the J chain of IgM [23]. Specifically, ERdj5 was shown to be required for reduction of interchain disulfide bonds in both substrates to ensure their efficient degradation. Mutational inactivation of the two most reducing thioredoxin domains in ERdj5 was subsequently shown to strongly influence the ERAD-enhancing activity of ERdj5 [18]. The efficiency of ERdj5 in catalyzing reduction-mediated ERAD has recently been shown to rely not only on the redox activity of the protein but also on its conformational plasticity [46].

ERdj5 has also been shown to promote reduction of the disulfide bonds connecting the VP1 subunits of the simian virus 40 (SV40) capsid in virus-infected CV-1 cells as a means to prepare the virus for translocation across the ER membrane, an obligate step for infection [47]. Previous work had demonstrated that ERp57 functions as an isomerase of VP1 interchain disulfides in HeLa cells [48]. ERdj5 and ERp57 were both found to collaborate with PDI 47, 48. Overall, reduction and/or disulfide-rearrangement of the SV40 capsid paves the way for structural rearrangements in the capsid, a process likely promoted by the chaperone function of PDI, which in turn generates a hydrophobic virus that can translocate to the cytosol assisted by various ERAD factors (Figure 3) [49].

PDI itself has also been shown to function as an ERAD/retrotranslocation reductase despite relatively unstable active-site disulfides. By analogy with the SV40 work described above, PDI reduces disulfides in murine polyomavirus in preparation for translocation of the virus to the cytosol [50]. PDI also reduces misfolded proinsulin [51] and the hedgehog precursor to allow retrotranslocation [52]. In addition, PDI has been implicated in reduction of the interchain disulfide between the A and B chains of the plant toxin ricin [53] (Figure 3). The PDI-released active A subunit is then retrotranslocated to the cytosol, where it exerts its deleterious function [54]. Another PDI family member, TMX1, has been shown to influence the retrotranslocation of ricin. TMX1 overexpression sensitized cells to the toxin, whereas downregulation protected cells from ricin intoxication [55]. It remains unknown whether TMX1 directly reduces ricin to allow retrotranslocation of the A subunit.

Finally, the recently discovered PDI family-member TXNDC11 is a potential ERAD reductase. This protein functions in glycoprotein ERAD and interacts with several well-characterized ERAD components [56]. The TXNDC11 active-site disulfide is very stable [56], even more so than active-site disulfides in ERdj5 [18]. However, the finding that TXNDC11 depletion stabilizes wild-type as well as the Cys-to-Ser mutant of the null Hong Kong variant of α1-antitrypsin to the same extent [56] indicates that the reductase activity of TXNDC11 may not target ERAD substrates, but instead another constituent of the ERAD machinery.

### The Role of NADPH-Dependent Reductases in ERAD

As discussed above, the molecule(s) that directly catalyze the reduction of active-site cysteines in PDIs such as ERdj5 are unknown. A role for cytoplasmic NADPH has been proposed, but it is tempting to speculate that there may also be an ER-localized source of reducing equivalents. Perhaps PDIs are maintained in a reduced state by an ER-localized reductase that is directly coupled to NADPH (i.e., a glutathione or thioredoxin reductase-like enzyme). However, no such activity has been identified in the ER [57]. Nonetheless, NADPH is recycled in this organelle – in a reaction catalyzed by hexose 6-phosphate dehydrogenase – and is used by 11β-hydroxysteroid dehydrogenase 1 to convert cortisone to cortisol [58].

One other potential NADPH-dependent ER-localized reductase is ERFAD (FOXRED2). This homolog of glutathione reductase has been implicated in ERAD 59, 60 and was shown to interact with a non-catalytic PDI-like protein, ERp90 60, 61. ERFAD is a flavoprotein that contains a consensus motif for NADPH binding, but does not contain cysteine residues at the positions of the active-site cysteines in glutathione reductase. ERFAD can be immuno-isolated with ERdj5, but no reductase activity towards protein disulfides has been demonstrated for ERFAD. Nevertheless, ERFAD does show reducing activity and is capable of reducing (and thus activating) the small-molecule prodrug SN29428 which targets tumor hypoxia [62]. How the small-molecule reducing activity of ERFAD, and the activity of other potential ER-localized NADPH-dependent enzymes, influence ER protein reduction and ERAD remains to be elucidated.

## Does Reduction of Oxidized Cysteines Play a Role in the Regulation of ER Protein Function?

Disulfide formation/reduction is important not only for protein folding and degradation but also for modulating protein activity. Efficient regulation of protein function through cysteine oxidation relies on the availability of oxidants and reductants to enable the effective transition between the oxidized and reduced cysteine forms. We outline here examples of how reductases affect redox regulation of ER-protein function.

### Reductase Control of Calcium Signaling

**Figure 4:**
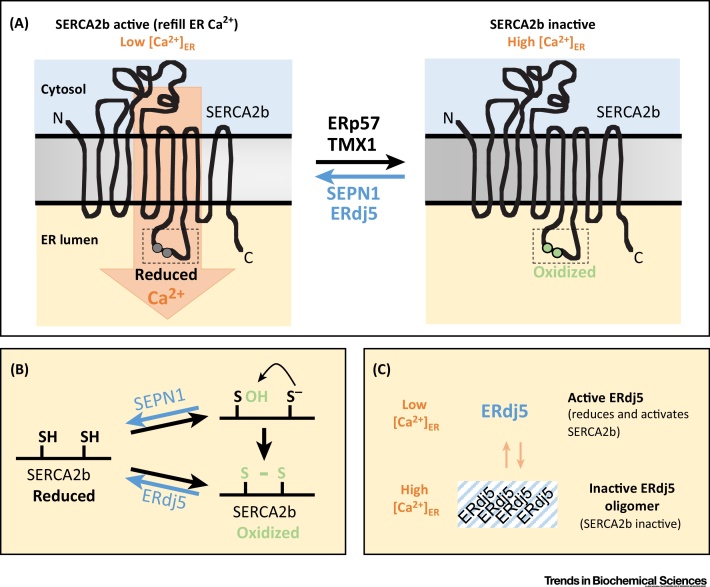
Reductases Associated with Redox-Regulation of SERCA2b. (A) Cysteines in a lumenally oriented loop of SERCA2b (circles highlighted within dashed box) can be oxidized by oxidoreductases (ERp57, TMX1) or small molecules (e.g., peroxide; not shown). Oxidation inactivates SERCA2b pump activity, limiting calcium import into the endoplasmic reticulum (ER). SERCA2b pump activity can be restored by the reductases ERdj5 and SEPN1. Reduction of the lumenal cysteines, and activation of SERCA2b activity, are associated with low ER calcium stores. (B) The two reductases SEPN1 and ERdj5 can reduce sulfenic acid-modified or disulfide-bonded SERCA2b cysteines, respectively. (C) ERdj5 reductase activity is calcium-dependent. Low ER calcium levels are associated with activation of ERdj5, whereas high ER calcium triggers oligomerization and inactivation of ERdj5.

A prototypical example of an ER redox-regulated protein is the sarco/endoplasmic reticulum calcium transport ATPase (SERCA), a calcium pump that imports calcium from the cytosol into the ER lumen. The activity of the SERCA2b subtype is negatively regulated by oxidation of a lumenal cysteine pair and is activated by reduction 63, 64. Formation/reduction of the regulatory SERCA2b disulfide occurs in a calcium-dependent manner, and redox regulation of SERCA2b activity regulates calcium import into the ER in response to changes in ER calcium levels (Figure 4A).

The PDI family members ERp57 and TMX1 have been implicated in the oxidation and inactivation of SERCA2b 65, 88. The reductases ERdj5 and SEPN1 are associated with the reduction, and thus activation, of SERCA2b 66, 67 (Figure 4). Disulfide-linked complexes between SERCA2b and ERdj5, or between SERCA2b and SEPN1, can be isolated from cells, suggesting redox-dependent interactions between these proteins and SERCA2b 66, 67. Stimulated calcium release from the ER is compromised in both ERdj5^−/−^ and SEPN1^−/−^ mouse embryonic fibroblasts, implying that both enzymes normally facilitate SERCA2b activation 66, 67. Knockdown of cellular ERdj5 resulted in increased levels of disulfide-bonded SERCA2b, further supporting a function for ERdj5 as a SERCA2b reductase [67]. Similarly, elevated levels of oxidized (sulfenylated) SERCA2b cysteines were observed in SEPN1 knockdown cells [66], suggesting that SEPN1 ordinarily serves to reduce sulfenylated SERCA2b (Figure 4B).

Calcium-dependent regulation of ERdj5 reductase activity has been proposed, centered upon the observation that ERdj5 undergoes oligomerization under conditions of high ER calcium concentrations (Figure 4C) [67]. This is reasoned to limit the interaction of ERdj5 with SERCA2b, maintaining SERCA2b in an inactive state. Conversely, ERdj5 dissociates into monomers at low calcium concentrations, enabling reduction of the SERCA2b regulatory disulfide and refilling of ER calcium stores [67]. Whether reduction of sulfenylated SERCA2b by SEPN1 is calcium-dependent is unexplored. One could envisage that SEPN1 reductase activity is constitutive, contrasting with the regulated activity of ERdj5. SEPN1 could serve to limit the overoxidation of the regulatory thiols to sulfinic or sulfonic acid, leading to irreversible inactivation of SERCA2b. For both ERdj5 and SEPN1 the initial reduction step would leave the enzymes oxidized and in need of reduction for recycling, necessitating a further reductase, so far unidentified.

### Reductases That Modulate the UPR

Changes in the ER-folding load are associated with redox events that modulate signaling through the UPR. Here the formation or reduction of disulfides within two UPR sensors (ATF6 and IRE1) modulates signaling. Two PDI family members, PDIR and P5, have emerged as disulfide exchange proteins that modulate redox changes in these UPR sensors.

During ER stress, the accumulation of unfolded proteins triggers IRE1 oligomerization and *trans*-autophosphorylation which ultimately drives UPR target gene induction [68]. Attenuation of IRE1 signaling, when stress subsides, is associated with the dissociation of IRE1 oligomers. It has been proposed that P5 reduces disulfide-bonded IRE1 oligomers to attenuate the UPR signal post-stress [69]. A mutant of P5 that stabilizes P5–substrate interactions can be trapped in a mixed disulfide with IRE1, implying electron exchange between the two proteins [69]. Moreover, overexpression of the P5 trapping mutant, but not a cysteine-less P5, accelerates dephosphorylation and inactivation of IRE1 [69]. Interestingly, preventing IRE1 disulfide formation by cysteine mutation also slowed attenuation of activated IRE1 [69]. Thus, although P5-mediated reduction of IRE1 may mediate attenuation, the breaking of the IRE1 disulfide appears to be insufficient to incite UPR decay. How P5 activity is controlled to facilitate the decline of the UPR signal post-stress is an open question.

In contrast to IRE1, ATF6 undergoes disulfide reduction during ER stress. Under non-stress conditions, ATF6 exists as a mixture of inter- and intramolecular disulfide-bonded species 70, 71. Disulfide reduction and disassembly during ER stress appear to be required, but are not sufficient, to mediate export of AFT6 to the Golgi [70]. Golgi localization allows proteolysis by the site-1 and site-2 proteases (S1P and S2P), which releases the cytosolic domain of ATF6 that then moves to the nucleus and acts as a transcriptional activator of UPR genes [68]. The PDI family member PDIR has been implicated in reduction of the ATF6 disulfides: silencing of PDIR limits oligomer dissociation and ER export of ATF6 under stress conditions [72]. Although direct electron exchange between ATF6 and PDIR has not been demonstrated, it seems likely that PDIR knockdown abolishes direct reduction of ATF6 disulfides by PDIR. Reduced ATF6 is a better substrate for S1P; it has been suggested that the less-efficient cleavage of oxidized ATF6 may benefit cells by allowing the retrieval of uncleaved ATF6 that has escaped from the ER (under non-stress conditions), thus limiting unsolicited UPR activation [70]. How ER folding stress is linked to an increase in PDIR-mediated reduction of ATF6 is unclear.

### Reductases That Influence Chaperone Function

Several ER molecular chaperones are prone to small-molecule oxidation, including the essential and abundant chaperones PDI and BiP. Nitrosylation and glutathionylation of the PDI active-site cysteines have been observed in cells, and oxidation can inhibit oxidoreductase activity 13, 73. A reductase that can reverse these adducts and restore PDI function has not been identified. BiP, an Hsp70 family member, contains a conserved cysteine within its ATPase domain, and this cysteine is both sulfenylated [77] and glutathionylated 6, 75, 76 in cells. Cysteine oxidation changes BiP activity, inhibiting normal ATPase activity but augmenting the interaction between BiP and polypeptide substrates 6, 77, 78. BiP modification is associated with conditions of ER oxidative stress, and an increase in BiP holdase activity is proposed to help to limit polypeptide aggregation during suboptimal folding conditions 77, 78. Recently an unexpected reductase has been identified for BiP, the protein Sil1, which can reverse a BiP cysteine–glutathione adduct and restore ATPase function to BiP [74].

Sil1 is one of two nucleotide exchange factors (NEFs) for BiP. NEF activity is associated with the C-terminal portion of Sil1, which binds to BiP and correspondingly weakens the contacts between BiP and its nucleotide [79]. The capacity for Sil1 as a reductant traces to a pair of cysteines within the N-terminus. When these cysteines are in the reduced form, recombinant Sil1 facilitates glutathione release from the BiP cysteine [74]. Cells lacking Sil1 (*sil1Δ*) show an accumulation of oxidized BiP and a slow reduction of oxidized BiP post-stress [74]. In contrast to the reductases discussed above, which are associated specifically with higher eukaryotic species, Sil1 is widely conserved. The activity of Sil1 as a reductase has been demonstrated to date only for yeast Sil1. What maintains Sil1 in a reduced state to allow BiP reduction is unknown.

## Concluding Remarks

We have outlined here many examples of proteins that require reduction of their thiol groups for correct folding, degradation, or regulation. Various types of cysteine-oxidation adducts can be generated in cells. The variability in cysteine species suggests that distinct reductases may be necessary to mediate the removal of specific modifications, and this could account (at least in part) for the description of multiple reductants that act on a given protein substrate (e.g., SERCA2b) and/or within a specific pathway (e.g., ERAD). The nature and components of reducing ER pathways are only now being identified, and several questions remain (see Outstanding Questions). Elucidating the specific mechanisms for reduction will be a particularly challenging question to address given the large number of substrates involved and the likely redundancy between individual pathways. We anticipate that future research will focus on the identification of the (missing) reducing pathway components, how reducing pathways are regulated, and how the redox balance within the ER is maintained to allow both oxidizing and reducing activities.

Outstanding Questions What are the relative roles of thiol reduction by (i) enzymatic catalysis, or (ii) directly by glutathione?

What are the identities of all the components of the reductive pathway(s) linking NADPH to thiol reduction in the ER?

What directly reduces the PDIs within the ER? Do ER-localized NADPH-dependent enzymes have a function in ER protein reduction?

How is specificity achieved between the oxidoreductases. Are some reductases selective for specific substrates, and/or are there general reductases that act on a broader range of substrates?

How is reductase activity regulated – spatially and/or temporally – in the case of regulatory reduction events?

What are the presumed reductase functions of the ER-localized enzymes SEPN1 and TXNDC11?

What is the influence of protein reduction on other post-translational modifications such as *N*-linked glycosylation?

There are several poorly characterized selenoproteins in addition to SEPN1 that reside within the ER. Do these proteins also act as reductases?
